# OpenThruster: An open-source, mostly 3D-printed thruster for marine vehicles

**DOI:** 10.1016/j.ohx.2025.e00680

**Published:** 2025-07-29

**Authors:** Milind Fernandes, Soumya Ranjan Sahoo, Mangal Kothari

**Affiliations:** a Department of Electrical Engineering, Indian Institute of Technology Kanpur, Kanpur, UP, India; b Department of Aerospace Engineering, Indian Institute of Technology Kanpur, Kanpur, UP, India

**Keywords:** 3D-printed, ASV, ROV, AUV, Open-source, Thruster

## Abstract

Thrusters are essential components of marine robotic vehicles for surface or underwater use. However, their high cost often makes them inaccessible to hobbyists, early-career researchers, and citizen scientists. With advancements in 3D printing, several do-it-yourself (DIY) thruster designs have emerged, allowing assembly using off-the-shelf components. However, most existing designs provide only printable files. These often lack detailed source information and, more importantly, performance data. This work presents the design, and an updated and expanded evaluation of the open-source OpenThruster project, with a focus on performance variability, fabrication methods, and dynamic modeling. OpenThruster is an open-source, low-cost, and mostly 3D-printed thruster for marine applications. The thruster itself is designed and simulated using open-source software. Performance evaluation is performed using off-the-shelf components and, wherever possible, open-source hardware. To ensure broad accessibility and long-term availability, we selected one of the most widely available drone motors and tested units with identical specifications from various vendors to assess consistency. Experimental validation involved a VESC6 driver board and bollard thrust measurements using a load cell setup in pool water. We also evaluated propellers produced, via three different 3D printing techniques. The thrusters consistently produced an average peak thrust of 18 N at 310 W, with fabrication costs kept under 500 INR (approximately $6). While thrust variation across ten motors from different vendors reached up to 11%, a one-way ANOVA test indicated no statistically significant difference between them. However, propellers made with different printing methods demonstrated significant differences in thrust output.

## Graphical abstract

**Figure:**
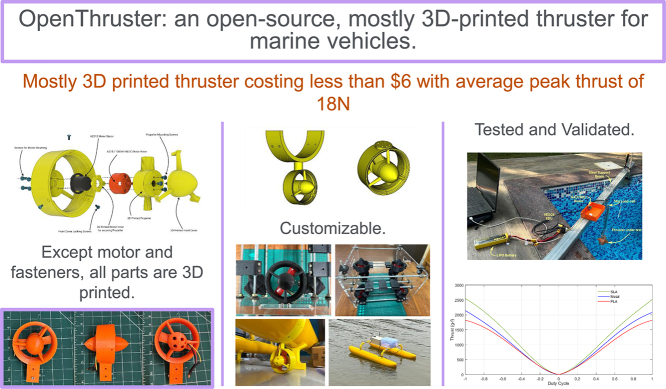

## Specifications table

**Table:** 

Hardware name	OpenThruster: Open source thruster for marine vehicles

Subject area	• Engineering and material science
	• Environmental, planetary and agricultural sciences
	• Educational tools and open source alternatives to existing infrastructure

Hardware type	• Electrical engineering and computer science
	• Mechanical engineering and materials science

Closest commercial analog	BlueRobotics T200 Thruster
Open source license	CERN-OHL-P
Cost of hardware	INR 500/$6
Source file repository	http://doi.org/10.17632/yfg487zbkp.1

OSHWA certification UID *(OPTIONAL)*	IN000064

## 1 Hardware in context

### 1.1 Motivation

Water bodies are fundamental to sustaining life on Earth. They can generally be categorized as unexplored, explored, or exploited. Regardless of category, studying them is essential for effective resource planning and management. Autonomous vehicles are now the cornerstone of aquatic exploration. They are used across a wide range of environments, including lakes, rivers, wells, ponds, seas, oceans, and even subglacial waters in polar regions [1]. However, these advanced tools come at a significant cost, with even the most basic platforms priced in the thousands of dollars. Many of the most exploited or polluted water bodies are found in low-income regions [2], [3]. This creates a pressing need for affordable solutions to support monitoring and restoration.

Aside from sensors, electronics, and power sources, the thruster is one of the most expensive components in a marine autonomous vehicle. This is primarily due to the need for brushless DC (BLDC) motors to be protected against water damage and corrosion. Specialized corrosion-resistant bearings or coatings are often required to ensure long-term reliability. For deep water operations, motors must be enclosed within oil-filled pressure housings, further increasing costs. Depending on their performance requirements, such specialized thrusters can cost several hundred dollars or more. Moreover, the marine robotics market is relatively niche compared to aerial drones, limiting the cost advantages from mass production.

However, the widespread availability of low-cost BLDC motors, mainly due to the proliferation of aerial drones, presents an opportunity for cost reduction in underwater thrusters. Unlike brushed motors, BLDC motors do not require a commutator and brushes, making them easier to waterproof using epoxy coatings. If an inexpensive motor is available, users can avoid costly coatings and bearings by relying on simple maintenance or easy replacement. By integrating these low-cost motors with 3D-printed components, a highly affordable thruster for marine autonomous vehicles can be developed.

This work builds upon our previous publication [4], where we introduced a low-cost, open-source 3D-printed thruster design. While the structural design of the thruster remains largely unchanged, the current study significantly expands upon performance characterization, introduces improved experimental methodology, and evaluates additional parameters such as motor variability, power supply effects, and manufacturing techniques. These enhancements aim to improve reliability and provide deeper insights for those intending to use or build upon the OpenThruster project. The present work thus represents a major step forward in making affordable underwater propulsion more accessible and better understood.

The next subsection provides an overview of research on 3D-printed thruster systems.

### 1.2 Literature review

Several open-source systems have been developed to gather data from water bodies [5], [6], [7], [8], [9], [10], [11]. These systems use vehicles that are either custom-built [5], [7], [9] or commercially available [8], [10], [11]. Even for custom-built platforms, thrusters are typically purchased off-the-shelf. In some cases, thrusters are assembled, but still use off-the-shelf propellers [7]. Among these, only [6] used a 3D-printed propeller. Thruster systems are often expensive, typically costing between $20 and $200.

Some 3D-printable thruster designs using quadcopter BLDC motors exist^1^ [12], [13], but many lack true open-source accessibility, and their source files are often unavailable for modification. When performance data is provided, it is usually limited to a few parameters or tests. Additionally, many designs use scaled-down versions of existing propellers rather than ones optimized for the motors, resulting in suboptimal performance.

Several studies in the literature have examined the design and analysis of propellers for underwater applications; however, only a limited number have investigated 3D-printed propellers or thrusters alongside corresponding performance evaluations. Among those that do, researchers have experimented with a range of materials including ABS [14], [15], [16], [17], [18], PLA [19], stainless steel [20], AlSi10Mg alloy [21], and Nylon 6 [22]. Most of these studies rely on industrial-grade 3D printers, which are often expensive, and only a few explicitly detail the fabrication methods or consider accessibility. Experimental validation is provided in a subset of works—for instance, [14], [21] employ large towing tanks, while [17], [18], [23] conduct tests in smaller tanks. Other studies focus on other factors such as material stiffness, surface finish, or the orientation of support structures and their effects on hydrodynamic performance [20], [21], [22].

A particularly detailed comparative evaluation is presented by Khaleed et al. [22], who assess multiple materials for underwater propulsion (UWP) applications. Their findings indicate that ABS and Nylon 6 perform best, exhibiting low Von Mises stress, minimal surface roughness, and good dimensional stability—attributes that make them suitable for high-performance use cases. In contrast, PLA demonstrated the highest stress and deformation, as well as poorer surface quality, rendering it the least preferred option. In terms of environmental exposure, polymer-based propellers showed negligible mass change in freshwater due to the counteracting effects of hygroscopic moisture absorption. Meanwhile, AA6061 experienced mass gain due to surface oxidation. In seawater, all materials exhibited mass loss, with AA6061 and PLA showing the most significant degradation. These results highlight the robustness of ABS and Nylon 6 for demanding underwater environments, while PLA may still be viable for less critical, general-purpose applications.

While these studies provide valuable insights, their differing objectives, experimental setups, and evaluation criteria make direct comparisons difficult. Nevertheless, a consolidated review — summarized in Table 1 — reveals several recurring themes. First, FDM-based 3D-printed propellers are most commonly reported, though propellers made using SLA or metallic processes typically outperform standard FDM materials like PLA or ABS. Second, although additively manufactured propellers demonstrate promising results in controlled environments, issues such as repeatability and material inconsistencies remain, particularly when using low-cost fabrication techniques. Third, only a few studies — most notably Kim et al. [17] — have attempted to develop a fully 3D-printed thruster, with the majority opting to use commercially available motors and housings. Notably, none of the reviewed works include cost analyses, and none have made their designs open-source.

To address the limitations identified in earlier research, our previous work [4] introduced a fully open-source thruster specifically designed for fabrication using a low-cost FDM 3D printer. Building on that foundation, the current study significantly extends the original effort by revisiting the design, offering detailed assembly instructions, providing guidance for potential modifications, and presenting a comprehensive performance evaluation. This updated work introduces several key advancements in performance characterization, methodological rigor, and practical usability:

1. **Motor Variability Study:** We assess thrust variation across identical motors from different vendors, addressing the prior assumption of uniform performance.
2. **Impact of Voltage Supply:** We examine how varying input voltage levels influence thrust performance—an aspect not explored in the previous work.
3. **Propeller Fabrication Techniques:** In addition to FDM, we compare propellers produced using MSLA and metal additive manufacturing. This highlights the influence of surface finish and blade stiffness on performance—factors not accounted for in the initial study.
4. **Dynamic Modeling:** We introduce models describing thrust as a function of PWM duty cycle, as well as angular velocity response. These models are useful for simulation and control design and were absent in the earlier publication.
5. **Enhanced Experimental Setup:** The current testing setup uses a larger pool with more stable water, reducing the effects of water circulation seen in the original inflatable pool setup. Additionally, the integration of the VESC6 driver enables real-time RPM logging, addressing key limitations of the original configuration.
6. **New Mounting Option:** A newly introduced mounting design supports ROV-style configurations, expanding the thruster’s applicability to a broader range of marine vehicles.

Overall, this work delivers a reproducible, low-cost, and accessible alternative to commercial thrusters, with the added benefit of being open-source to support broader adoption, customization, and collaborative improvement.

**Table 1:** Literature review summary.

Ref.	AM thruster	AM propeller	Process	Material	Size(Dia.)	Appl.	Designed thrust	Exp.	Cost	Open source
[14]	No	Yes	FDM	ABS	0.625 m	AUV	75 N	No	N.S.	Only design code
[15]	No	Yes	FDM	ABS	N.S.	AUV	55–75 N	No	N.S.	No
[16]	No	Yes	SLA	High temp. resin	N.S.	N.S.	60 N	Yes	N.S.	No
[17]	Yes	Yes	FDM	ABS	0.033 m	AUV	0.4 N	Yes	N.S.	No
[18]	No	Yes	FDM	ABS	0.22–0.25 m	BOATS	300 N	No	N.S.	No
[19]	Yes	Yes	FDM	PLA	N.S.	AUV	N.S.	Yes	N.S.	No
[20]	Yes	Yes	FDM/SLM	ABS/Metal	N.S.	ROV	N.S.	No	N.S.	No
[21]	No	Yes	FDM/SLS	Z-ABS/Z-UltraT/AlSi10Mg	N.S.	N.S.	N.S.	Yes	N.S.	No
[22]	No	Yes	FDM/Cold forging	PLA/Nylon/ABS/Al6061	N.S.	N.S.	N.S.	Yes	N.S.	No
[23]	Yes	Yes	N.S.	N.S.	N.S.	ROV	30 N	No	N.S.	No

N.S. - Not specified. AM - Additive manufactured. Appl. - Application area. Exp. - Experimental

### 1.3 Design goals

Due to the absence of a readily modifiable open-source thruster design suitable for our marine vehicle, the primary goal of this work was to develop an affordable thruster that could be fabricated using a low-cost FDM 3D printer. This constraint required that the only off-the-shelf component — the BLDC motor — also be both low-cost and widely available. This decision was driven by the intent to democratize access to underwater propulsion technology and enable rapid prototyping and customization for hobbyists, researchers, and developers operating with limited resources.

The second major objective was accessibility. By relying primarily on 3D printing for fabrication, the design circumvents trade barriers and import restrictions, making the thruster more accessible globally. This accessibility is further enhanced by the choice of a motor that is commonly and easily sourced. However, this choice introduces variability in performance across motors from different vendors, as there is no guarantee of uniformity. The analysis of this variability constitutes one of the key contributions of this work.

In line with the accessibility goal, this project adopted an open-source philosophy from the outset. All design, simulation, and testing processes were carried out using freely available tools whenever possible. This not only reduced the overall development cost but also promoted transparency and reproducibility. An additional benefit of the accessibility-focused design is that the thruster is inherently easy to service and repair.

Regarding performance, the target was set at a modest maximum thrust of 20 N using a 12 V power supply. This benchmark was chosen because it approximates the average thrust of the BlueRobotics T100 thruster and is comparable to that of the SeaBotix BTD150, a widely used model in earlier ROVs. Furthermore, the thruster was designed to provide nearly symmetrical thrust in both forward and reverse directions, making it suitable for use in both surface and underwater remotely operated vehicles (ROVs).

Durability and reliability were intentionally given lower priority. High durability typically requires manufacturing via injection molding in ABS or CNC machining in metal—both of which are prohibitively expensive for low-cost designs. Similarly, reliability often demands tight manufacturing tolerances and rigorous quality control, which are not feasible when using generic BLDC motors from multiple vendors. These trade-offs are in direct conflict with the goals of affordability and accessibility.

**Fig. 1:**
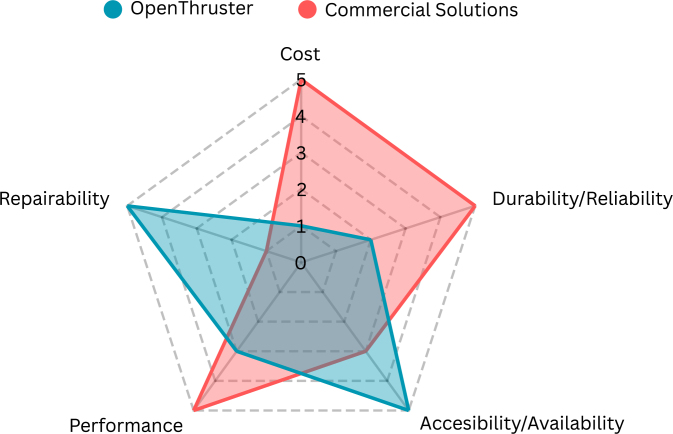
Radar chart of design goals.

A summary of the design goals and the challenges addressed in this work is presented in the radar chart shown in Fig. 1.

## 2 Hardware description

**Fig. 2:**
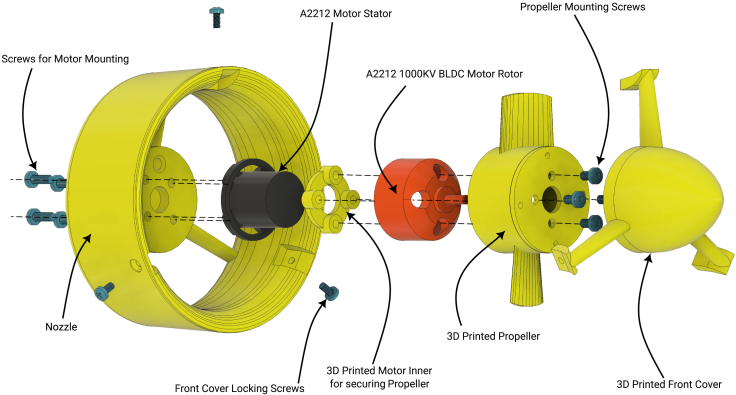
Rendered exploded view of the thruster design.

In this section, we briefly describe the design of the OpenThruster and its main components. The thruster consists of four primary elements: a BLDC motor, a propeller, a nozzle/duct, and various screws for assembly. An exploded view is shown in Fig. 2.

**Fig. 3:**
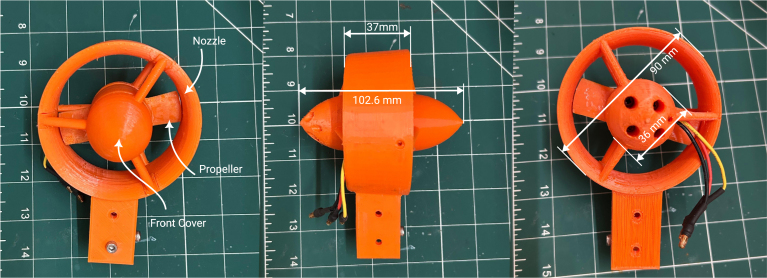
An assembled thruster (R-L: Front, side and back view).

Among these components, two are 3D printed: the nozzle/duct and the propeller assembly. The nozzle/duct includes both the nozzle and a front cover, while the propeller assembly includes the propeller and a part referred to as the motor inner. In total, the design includes four distinct 3D-printed parts. The motor used is an A2212 1000 KV BLDC model—an inexpensive (less than $5), widely available motor commonly used in quadcopters. Its nominal specifications are listed in Table 2. The *motor inner* part secures the propeller to the motor. The front cover aids water flow through the nozzle by reducing drag and preventing flow separation at the hub. A fully assembled view of the thruster is shown in Fig. 3.

**Table 2:** A2212 1000 KV BLDC motor parameters.

Parameter	Value	Parameter	Value
KV	1000 RPM/V	Weight	52.7 g
$I_{max}$	13 A for 60 s	Size	28 × 28 mm
$I_{NoLoad}$	0.5 A @ 10 V	Max. efficiency	80%
Battery cells	3S	Poles	14
Max. power	150 W	Resistance	0.090 Ω

### 2.1 Propeller and nozzle design

The propeller is designed to mount directly onto the rotor (or bell/housing) of the BLDC motor, following an approach similar to the Blue Robotics T200 thruster. Due to the limited torque capability of the A2212 1000 KV motor, a two-blade configuration was chosen to optimize performance. Based on the motor’s dimensions, the propeller root diameter was set to 36 mm. The design also assumes a maximum vehicle speed of 1.5 m/s.

For the propeller design, we used OpenProp v3.3.4 [24], an open-source, MATLAB-based tool. (MATLAB was the only proprietary software used in this study.) The blades are based on the NACA 001612 profile, a symmetric airfoil that helps achieve a balanced thrust profile in both forward and reverse directions, aligning with our design goals. The final CAD model of the propeller was created using FreeCAD,^2^ an open-source CAD tool. The model was developed using data points exported from OpenProp. The final specifications of the propeller are listed in Table 3.

Underwater thrusters are generally categorized as either nozzle-equipped or nozzle-less. OpenThruster uses a 3D-printed nozzle to enhance thrust output, following the nozzle-equipped approach. Duct No. 37 from the Wageningen ducted propeller series [25] was chosen over duct No. 19A for its ability to provide more symmetrical thrust during both forward and reverse operation. These ducts are well-established in marine propulsion literature. For completeness, the ordinates for Duct No. 37, reproduced from [4], are listed in Table 4. For more details on the propeller design methodology and the use of OpenProp, readers are referred to our previous work [4].

The thruster design, particularly the nozzle, can be easily modified in CAD software to fit specific vehicle requirements. One such modification is illustrated in Fig. 3, in comparison with the original design shown in Fig. 2. This version allows the thruster to be mounted onto a standard 20 mm aluminum extrusion, making it suitable for both bench testing and integration into a vehicle. A comparison of our design with other low-cost commercial and partially open solutions is provided in Table 5.

**Table 3:** 3D-printed propeller specifications [4].

Parameter	Value
Diameter	72 mm
Hub diameter	36 mm
Foil shape	NACA 001612
Chord length	20 mm
Geometric pitch	45.43 mm
Pitch at root	21.32°
Pitch at tip	10.86°

**Table 4:** Ordinates for duct no. 37 [4].

x/D	0 (LE)	0.0125	0.025	0.050	0.075	0.1	0.15	0.2	0.25
$y_{i}$/D	0.1833	0.15	0.131	0.1	0.079	0.0611	0.0360	0.02	0.01
$y_{u}$/D	0.1833	0.213	0.217	0.216	Straight line				

									

0.3	0.4	0.5	0.6	0.7	0.8	0.9	0.95	1 (TE)
0.004	0	0	0	0.002	0.011	0.038	0.066	0.1242
Straight line							0.16	0.1242

								

${}^{*}i$ and u denote the inner and upper ordinate. is the diameter. LE and TE denote leading and trailing edges.

**Table 5:** Comparison of low cost thrusters.

Parameter	This work^a^	Blue robotics	APISQUEEN	ROV	Laidani et. al.
		T200	U01^b^	Thruster^c^	[26]
Max. thrust F/R (N)	18/17.7	51.5/40 (@16 V)	11.8/7.8 (@12 V)	46.8/41	12.25/10.78
Max. current (A)	26.8	24 (@16 V)	17 (@16 V)	25	21
Operating voltage (V)	12	7–20	10–16	12	12
Max. power (W)	312	390 (@16 V)	390	300	250
RPM range	500–4900	300–3500	–	–	700-15000
Weight in air (g)	140	344	178	190	152
dimensions (mm)	103 × 90	113 × 100	72 × 75	118 × 120	98 × 70
Cost	$6	$200	$16	$50	$45

a PLA propeller.

b https://www.underwaterthruster.com/

c https://web.archive.org/web/20250318210129/http://rovthruster.com/

### 2.2 Electrical connections

**Fig. 4:**
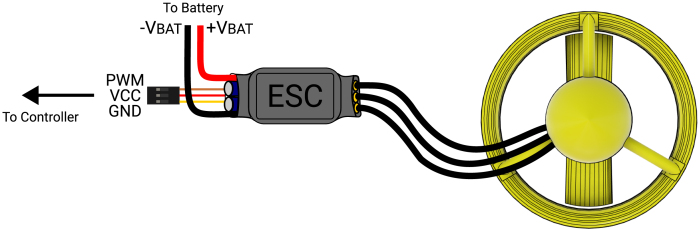
Electrical connections of the thruster with ESC.

The wiring diagram is shown in Fig. 4. The setup is straightforward: the electronic speed controller (ESC) connects to the battery, the main controller, and the BLDC motor on the thruster. The ESC should support a peak current of at least 30 A at 12 V. Depending on the application and manufacturer capabilities, the ESC can be configured for either unidirectional or bidirectional operation.

### 2.3 Applications

**Fig. 5:**
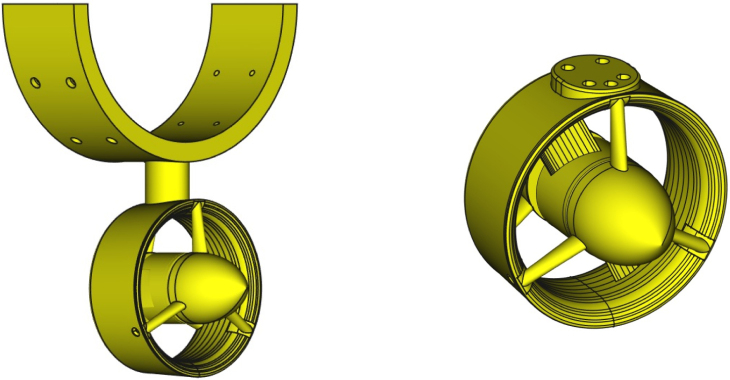
Modifications to the nozzle design.

In addition to being ultra-low cost, the developed thruster offers several other advantages. Because the nozzle design is fully open-source, it can be modified to meet specific application needs—unlike many proprietary, off-the-shelf solutions. One example of this flexibility is shown in Fig. 5.

**Fig. 6:**
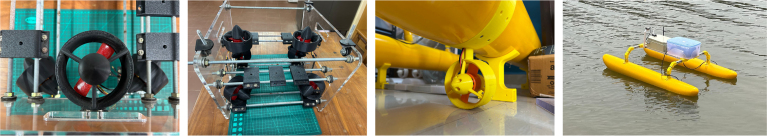
Implementations of modified thruster designs, ROV (L) and ASV (R).

The left image illustrates a nozzle extension tailored to match the hull shape of a surface vehicle. The right image shows a modification that allows the thruster to be mounted on a remotely operated vehicle (ROV). Both configurations are shown in Fig. 6.

The cost-effectiveness of the developed thruster becomes especially apparent in ROV applications. Since ROVs typically require six to eight thrusters, expenses can escalate quickly. In contrast, using the design presented in this work, eight thrusters can be fabricated for just $48.

## 3 Design files summary

The complete design files are available at http://doi.org/10.17632/yfg487zbkp.1. The location of the file in Table 6 below indicates the folder within the repository.

1. Motor_inner_Helper: CAD file of 3D printed part used to install the motor inner within BLDC motor rotor.
2. Motor_Inner_v2: CAD file of motor inner 3D printed part used to fasten the propeller onto the BLDC rotor.
3. Prop-CCW & Prop-CW: CAD file of counterclockwise or clockwise propeller.
4. Thruster_Assembly: CAD file of entire thruster assembly.
5. Motor_inner_Install_Helper: stl format file of 3D printed part used to install the motor inner within BLDC motor rotor.
6. Motor_Inner: stl format file of motor inner.
7. Nozzle_Front_cover: stl format file of nozzle front cover.
8. Prop-CCW & Prop-CW: stl format file of counterclockwise or clockwise propeller.
9. Thruster_Nozzle: stl format file of only the thruster nozzle.

In addition to the design files, the repository includes a folder containing the OpenProp software, the datasheet for the BLDC motor, propeller data used for the FreeCAD design, assembly instructions, and details of the testing instrumentation setups. A pre-sliced .3mf file is also provided in the STL folder for ease of 3D printing.

**Table 6:** Design file summary table.

Design filename	File type	Open source license	Location of the file
Motor_inner_Helper	CAD (FCstd)	CERN-OHL-P	/FreeCad_v_1.0_Source_Files
Motor_Inner_v2	CAD (FCstd)	CERN-OHL-P	/FreeCad_v_1.0_Source_Files
Prop-CCW	CAD (FCstd)	CERN-OHL-P	/FreeCad_v_1.0_Source_Files
Prop-CW	CAD (FCstd)	CERN-OHL-P	/FreeCad_v_1.0_Source_Files
Thruster_Assembly	CAD (FCstd)	CERN-OHL-P	/FreeCad_v_1.0_Source_Files
Motor_inner_Install_Helper	CAD (stl)	CERN-OHL-P	/STL
Motor_Inner	CAD (stl)	CERN-OHL-P	/STL
Nozzle_Front_cover	CAD (stl)	CERN-OHL-P	/STL
Prop-CCW	CAD (stl)	CERN-OHL-P	/STL
Prop-CW	CAD (stl)	CERN-OHL-P	/STL
Thruster_Nozzle	CAD (stl)	CERN-OHL-P	/STL

## 4 Bill of materials summary

The Bill of Materials (BoM) in Table 7 lists the cost of a single thruster, excluding the Electronic Speed Controller (ESC). The cost of the 3D-printed parts is estimated based on the weight of each individual component. A 1 kg roll of PLA filament is priced at approximately INR 1000 ($12). Please note that the cost of the 3D printer itself or any associated usage charges are not included in this calculation.

A 30A BLDC ESC can be purchased for anywhere between INR 350 ($5) and INR 1600 ($19), depending on the brand and included features. Alternatively, instead of using FDM printing with PLA, the propeller can be manufactured using a Masked SLA (MSLA) process with ABS-like resin or fabricated from metal alloy via powder sintering. The MSLA process produces a smoother blade finish and eliminates the need for sanding, thereby reducing post-processing time and enhancing thruster efficiency. However, this method is more expensive than FDM printing. Metal propellers made through powder sintering offer greater durability and stiffness, but they are the most costly to manufacture and are significantly heavier. Users can select the most appropriate option based on their specific cost-performance trade-offs and application requirements. A comparative evaluation of propellers produced using these three methods is presented in Section 7.2.3. For reference, the bill of materials for the ESC and the resin/metal 3D-printed propellers is provided in Table 8.

**Table 7:** Bill of materials table.

Designator	Component	Number	Cost per unit - currency	Total cost - currency	Source of materials	Material type
Motor_inner_Install_Helper	Motor inner helper	1	INR 5	INR 5	Amazon.in	Polymer
Motor_Inner	Motor inner	1	INR 1	INR 1	Amazon.in	Polymer
Nozzle_Front_cover	Nozzle front cover	1	INR 14	INR 14	Amazon.in	Polymer
Prop-CCW, Prop-CW	Propeller	1	INR 12	INR 12	Amazon.in	Polymer
Thruster_Nozzle	Nozzle/Duct	1	INR 53	INR 53	Amazon.in	Polymer
BLDC Motor	A2212 1000 KV	1	INR 360	INR 360	Amazon.in	Metal
Front Cover Screws	M2.5 × 5 mm	3	INR 15	INR 15	Local hardware	Metal
Motor Mount Screws	M3 × 8 mm	4	INR 8	INR 8	Local hardware	Metal

**Table 8:** Bill of materials table for other fabrication methods.

Designator	Component	Number	Cost per unit - currency	Total cost - currency	Source of materials	Material type
Prop-CCW, Prop-CW	Propeller	1	INR 1300	INR 1300	makenica.com	Polymer
Prop-CCW, Prop-CW	Propeller	1	INR 5800	INR 5800	PCBway.com	Metal
ESC	Emax BLHeli 30A	1	INR 1600	INR 1600	robu.in	Other

## 5 Build instructions

To assemble the thruster, all 3D-printed parts must first be printed using PLA or another suitable material such as ABS or PETG. Between PLA and ABS, ABS offers higher stiffness and exhibits lower mass loss when submerged in water [22]. However, ABS requires an enclosed printer, higher printing temperatures and proper ventilation. PETG, on the other hand, has better chemical resistance, tensile strength, and durability than PLA, but it is known to be hygroscopic. For all parts except the propeller, the recommended print settings are a 0.2 mm layer height and 15% infill. For the propeller, it is advisable to use the smallest possible layer height supported by the printer, with supports enabled. In this work, the adaptive layer height feature in PrusaSlicer^3^ was used at a quality setting of 0.1. All parts were printed using a Creality Ender 3 V2^4^ 3D printer.

**Fig. 7:**
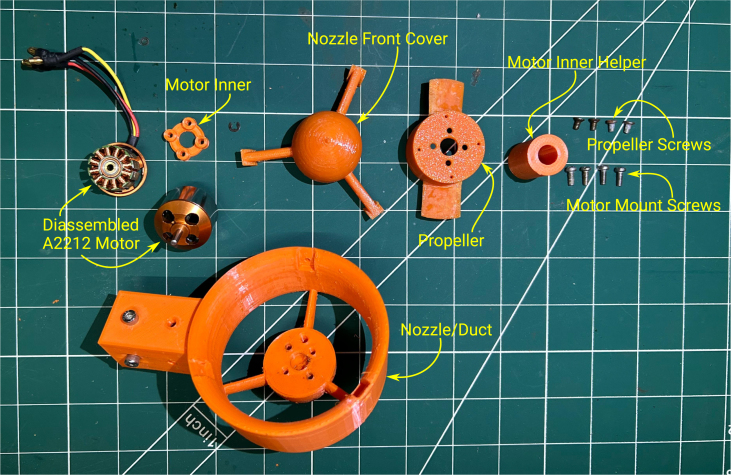
Parts required to assemble the thruster.

All components required for assembling the thruster — except the nozzle front cover screws — are shown in Fig. 7. It is important to ensure the 3D printer is properly calibrated for tolerances using calibration prints. This helps ensure that the parts are printed to the correct specifications.

The expected print time for one complete set of 3D-printed parts is approximately 12 to 14 h. Removing and cleaning the supports takes about 30 min, while sanding the propeller blades requires an additional 15–20 min. Once all components are ready, the final assembly of the thruster takes about 10–15 min. In total, the entire process — from printing to final assembly — requires approximately 13 to 15 h per thruster.

**Fig. 8:**
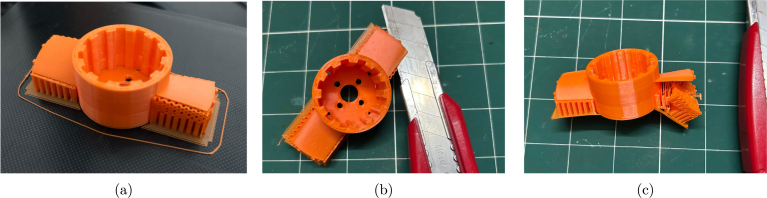
Removal of support structures from the propeller: (a) FDM 3D printed propeller on the print bed, (b) inserting a thin knife between the blade and the support structure, and (c) support structures successfully separated from the propeller blades.

The steps to assemble the thruster are as follows:

Step 1: The first step is to prepare the propeller. When the propeller is 3D printed using an FDM printer, the support structures must be removed. Fig. 8(a) shows the FDM 3D printed propeller with supports. Once the print is removed from the print bed, the supports must be carefully separated to avoid damaging the propeller blades. To do this, gently insert a thin knife between the support structure and the propeller blade, as shown in Fig. 8(b). Once the knife is about halfway along the blade, gently push it away from the blade. The support material should then separate, as shown in Fig. 8(c). For the other 3D printed parts, the supports can be removed like any typical print, and no special care is required.

**Fig. 9:**
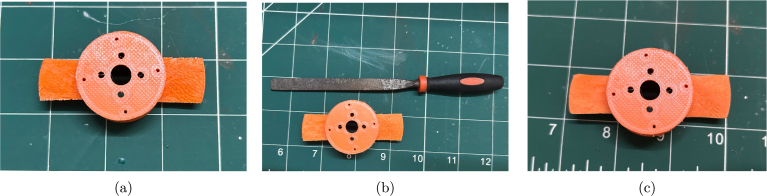
Preparation of the propeller: (a) after support removal, (b) after filing, and (c) after sanding.

This support removal process typically leaves a rough surface on the propeller blades, as shown in Fig. 9(a). To smooth the blades, begin by gently filing them with a thin file, as illustrated in Fig. 9(b). Next, sand the blades using 120-grit sandpaper, followed by 240-grit for a finer finish. The fully prepared propeller is shown in Fig. 9(c).

Optionally, the propeller blades can be coated with a thin layer of spray lacquer or epoxy resin. This helps extend their lifespan and improves efficiency by reducing surface friction.

**Fig. 10:**
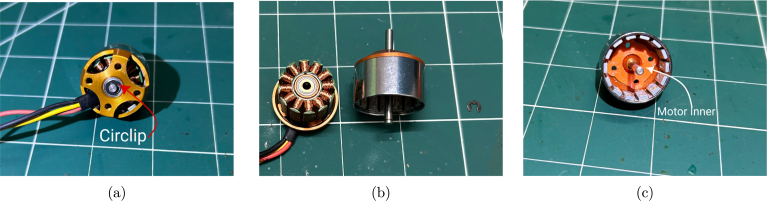
Motor preparation: (a) removal of the circlip as indicated, (b) disassembled BLDC motor, and (c) motor inner placed inside the rotor.

Step 2: The next step is to disassemble the A2212 BLDC motor by removing the circlip that holds the stator and rotor together, as shown in Fig. 10(a). Once the circlip is removed, gently pull apart the two parts of the motor. This may require some effort, as the internal magnets are quite strong. The disassembled motor is shown in Fig. 10(b).

For added protection, the stator windings can optionally be coated with oil-based paint or enamel. After this, insert the 3D-printed motor inner part into the rotor housing, as shown in Fig. 10(c). Ensure that the motor inner part fits snugly and is flush with the rotor housing.

**Fig. 11:**
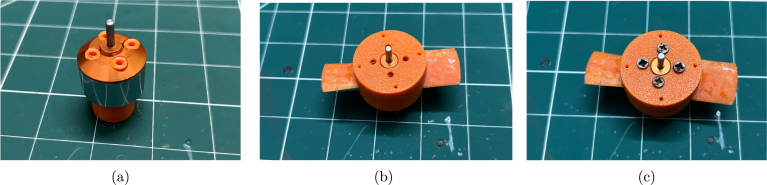
Assembly of the propeller on the rotor: (a) using the motor inner install helper, (b) aligning the screw holes, and (c) the final assembled propeller attached to the rotor.

Step 3: Next, secure the motor inner using the 3D-printed motor inner helper, as shown in Fig. 11(a). Then, place the propeller onto the motor rotor and align the screw holes. Use the four Phillips screws supplied with the motor to fasten the propeller to the rotor.

Be careful not to over-tighten the screws, as they grip into the holes in the motor inner part. Over-tightening can strip the plastic, requiring the motor inner to be replaced. The motor inner install helper keeps the motor inner in place and level during this step. The final assembly of the propeller attached to the rotor is shown in Fig. 11(c).

Step 4: Reinstall the rotor onto the stator and ensure it rotates freely without any friction. If there is any resistance, it may indicate that the stator coil is rubbing against the motor inner. In that case, remove the propeller, motor inner and repeat steps 2 and 3. Tighten the screws while applying a small amount of force that pushes the motor inner install helper against the rotor housing, which may help correct alignment.

**Fig. 12:**
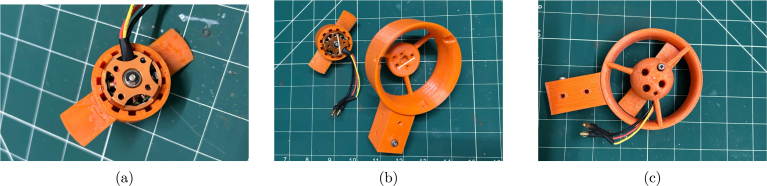
Assembly of the propeller and motor on the nozzle: (a) reinstalling the circlip, (b) aligning the screw holes, and (c) affixing the motor mounting screws.

Once the rotor spins freely, reinstall the circlip on the motor shaft, as shown in Fig. 12(a). Next, align the motor mounting holes with those on the 3D-printed nozzle. Be careful, as the two pairs of holes are spaced at different distances. Use the motor mounting screws to secure the motor onto the nozzle, as shown in Fig. 12(c).

**Fig. 13:**
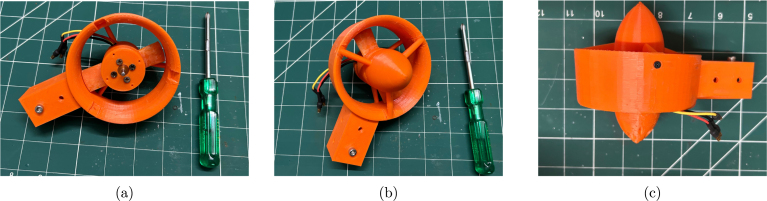
Assembly of the front cover on the nozzle: (a) nozzle with motor installed, (b) installing the front cover, and (c) affixing the front cover screws.

Step 5: The final step is to install the nozzle front cover. The front nozzle cover is fixed with the front cover screws, as shown in Fig. 13(c).

## 6 Operation instructions

The electrical connections for the assembled thruster with the controller of choice are shown in Fig. 4. Depending on the electronic speed controller used, an appropriate PWM range will need to be configured in the controller of choice. The configuration of the thruster for bidirectional or unidirectional operation will depend on the programmability of the ESC. Additionally, ESC configuration varies by brand and should be detailed in the respective datasheet.

One important precaution is to insulate the connection between the motor and the ESC. This can be done using waterproof heat shrink tubes or normal heat shrink tubes and sealing them with super glue.

If the thruster is used in fresh water, it should be properly dried after each use. It is also recommended to apply a penetrating oil, such as WD-40, to prevent rust on the motor and screws. When used in saltwater, the thruster should be rinsed in fresh water (for example, in a tub) to remove any salt buildup. After rinsing, applying penetrating oil will help prolong the thruster’s lifespan.

## 7 Validation and characterization

This section presents the most important contribution of this work. While open-source thruster designs exist, they often lack thorough performance validation. The designed thruster was validated through both simulations and experiments, which are detailed in the following sections.

### 7.1 Simulation

**Fig. 14:**
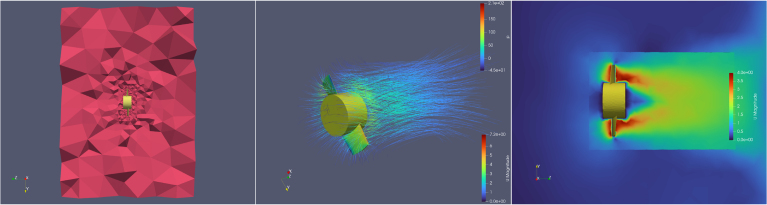
CFD simulation: meshing result, velocity streamlines and velocity distribution at 5000 RPM.

The designed propeller was simulated using a workflow composed entirely of open-source software. The model was meshed using Salome,^5^ an open-source tool for meshing complex geometries. The meshed model was then simulated in the open-source computational fluid dynamics software OpenFOAM.^6^ The resulting mesh, along with the streamlines and velocity distribution, is shown in Fig. 14.

**Fig. 15:**
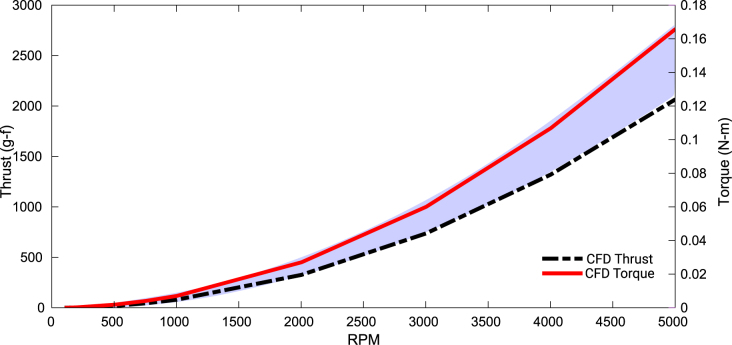
Thrust and torque curves from CFD simulations. The shaded region indicates experimental thrust measurements (all motors with MSLA resin propeller).

The thrust and torque versus speed are plotted in Fig. 15. The shaded region represents experimental thrust data presented in later sections. As seen, the thrust curve from the simulation forms a lower bound to the experimental data. This not only validates both the simulation and the experiments, but also indicates the additional thrust contribution of the nozzle that is not considered in simulation.

### 7.2 Experimental validation

**Fig. 16:**
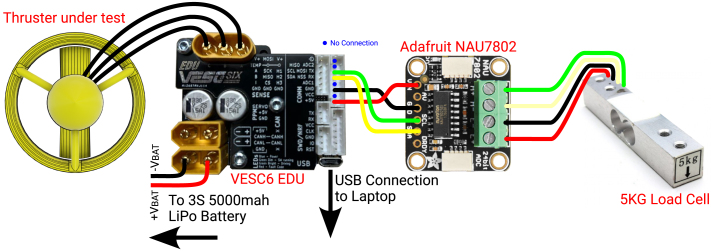
Instrumentation setup for experimental validation.

The instrumentation setup used for experimental validation is shown in Fig. 16. This configuration utilizes an open-source VESC6 EDU [27] motor controller board to control the motor and log its current, voltage, and electronic RPM. The actual RPM is calculated by dividing the electronic RPM by the number of pole pairs (seven in this case). Load cell data is also logged into the VESC6 board via an NAU7802 breakout board from Adafruit.^7^ The system is powered by a three-cell LiPo battery. The VESC6 communicates with a laptop running VESC-Tool software for configuration, control, and data display. This was the third iteration of the experimental setups; the previous two used Arduino or Pixhawk boards. The final choice of VESC6 was based on its ability to capture fine-grained data without requiring additional code.

**Fig. 17:**
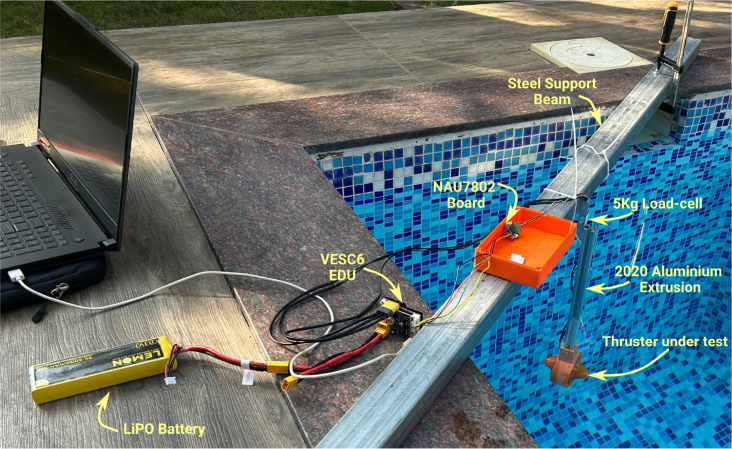
Pool testing setup with VESC6.

The thruster was tested in a pool to minimize the effect of circulating water on its performance. The physical setup is shown in Fig. 17. The thruster is mounted at one end of a 430 mm long, 2020 aluminum extrusion, with the other end fixed to a 5 kg load cell. This load cell is, in turn, attached at the midpoint of a 2 m-long, 60 mm × 40 mm hollow rectangular steel tube using two M4 × 10 mm screws. The steel tube is clamped onto the lip of the pool as shown. The load cell was initially calibrated with known weights, and the calibration constant is configured in a LISP script running on the VESC6 EDU board. The script is provided in the linked data repository.

**Fig. 18:**
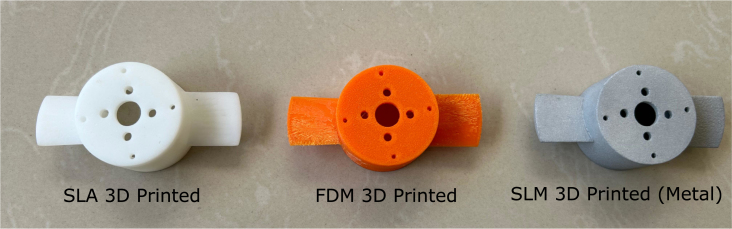
Propellers fabricated using three different 3D printing techniques.

The primary goal of the experimental validation was not only to gather performance data for the thruster but also to assess variations when motors are sourced from different vendors and propellers fabricated using different 3D printing techniques. For this, we procured ten A2212 1000 KV motors from different vendors and fabricated propellers using three 3D printing methods: FDM, MSLA, and SLM. The three propellers are shown in Fig. 18.

The first propeller on the left was produced via SLA 3D printing with ABS-like resin. It has the smoothest surface finish and weighs approximately 11.9 g. The middle propeller was made using FDM on a budget Creality Ender 3 printer with PLA filament; it weighs the same as the SLA-printed propeller. The third propeller, on the right, is made of aluminum alloy (AlSi10Mg) and fabricated using the SLM process by PCBWay. This propeller has a medium surface finish and weighs 30.8 g, nearly three times as much as the other two.

#### 7.2.1 Testing different motors

**Fig. 19:**
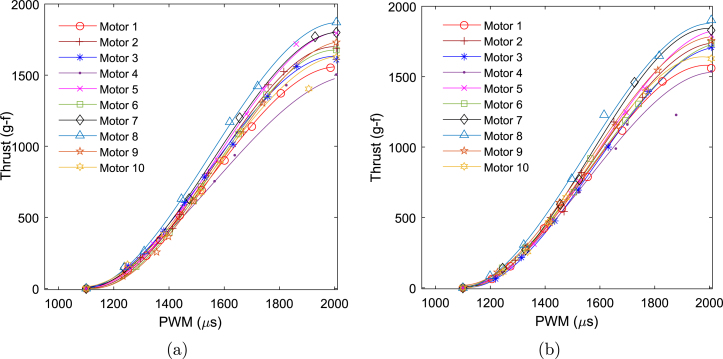
Thrust versus PWM for all 10 motors: (a) reverse direction and (b) forward direction.

**Fig. 20:**
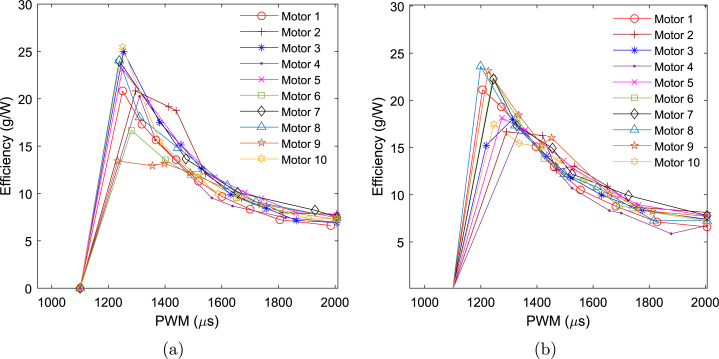
Efficiency versus PWM for all 10 motors: (a) reverse direction and (b) forward direction.

The measured thrust and efficiency plots for ten A2212 motors sourced from different vendors are shown in Fig. 19, Fig. 20. The curves in Fig. 19 represent thrust data interpolated using third-order polynomial curve fitting, while the markers indicate the actual collected data points. The plot reveals significant variation in thrust generated by motors from different vendors at a given PWM value. For instance, Fig. 19 shows a peak thrust variation of nearly 360 g-f between the best- and worst-performing motors. Considering a nominal thrust of 1700 g-f in both forward and reverse directions, this corresponds to approximately ±11% variation from the nominal value. Notably, a deadband region is observed between 950 and 1100 μs PWM values in both cases. The average thrust coefficient across the ten motors is calculated to be $k_{t}=8.5104\times 10^{-5}$, with a standard deviation of $2.8514\times 10^{-6}$. From Fig. 20, it is apparent that the average efficiency of all motors remains below 10 g/W at the highest PWM values.

**Fig. 21:**
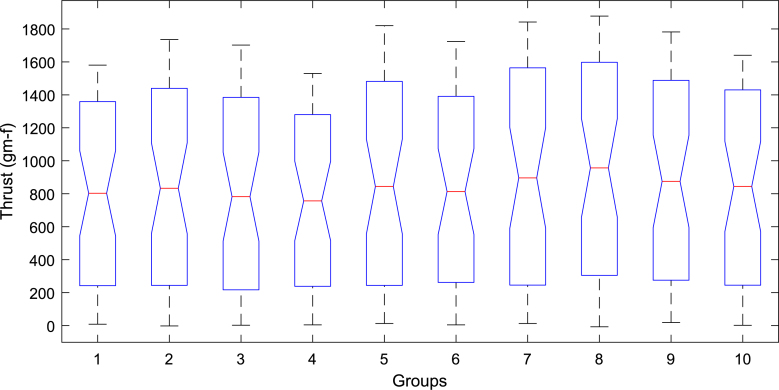
One-way ANOVA results of ten motors. (For interpretation of the references to color in this figure legend, the reader is referred to the web version of this article.)

A one-way ANOVA was conducted to evaluate the thrust performance across a set of ten motors. The results are visualized in the notched boxplot shown in Fig. 21. In this figure, the blue boxes represent the interquartile range (IQR), while the red lines denote the medians. The whiskers extend to the minimum and maximum values within 1.5 times the IQR from the first and third quartiles, or to the actual data limits when no outliers are present. The notches provide an approximate 95% confidence interval for the median; overlapping notches between groups suggest that the differences in medians are not statistically significant. The detailed ANOVA results are summarized in Table 9. The analysis yielded a P-value of 0.9437, which is substantially greater than the conventional significance threshold of 0.05. Therefore, the null hypothesis—that all group means are equal—cannot be rejected. This conclusion is consistent with the visual interpretation of the notched boxplot, where all groups exhibit overlapping notches. These findings indicate that the observed variation in thrust (approximately ±11%) across the ten motors is not statistically significant and can be considered negligible for practical purposes.

**Table 9:** One-way ANOVA statistics for ten motors.

Source	SS	df	MS	F	P
Groups	1.27635e＋06	9	141 816.4	0.38	0.9437
Error	1.6711e＋08	450	371 356.4		
Total	1.68387e＋08	459			

#### 7.2.2 Effect of supply voltage

**Fig. 22:**
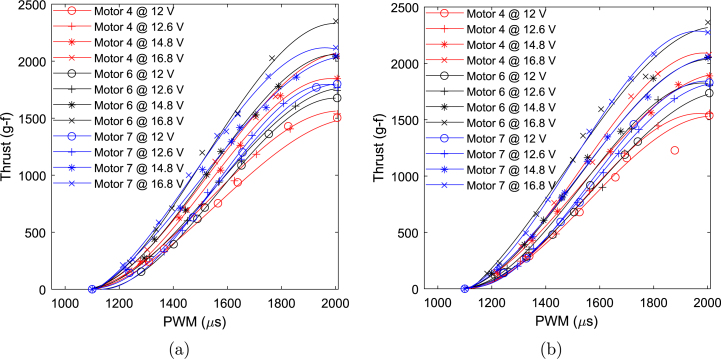
Thrust versus PWM at different voltage values: (a) reverse direction and (b) forward direction.

The tests for the thruster’s performance at different voltages were conducted using a random subset of motors—specifically motors 7, 6, and 4. These were tested at supply voltages of 12, 12.6, 14.8, and 16.8 V, powered by a lab bench power supply. These voltages correspond to typical values for 3S and 4S LiPo battery cells, including average, nominal, and peak voltages. The thrust versus PWM for the three motors at different supply voltages is shown in Fig. 22. The figure shows that thrust increases with supply voltage for a given PWM across all motors. The results indicate that using a 16.8 V supply increases thrust by up to 30% compared to 12 V, while a 14.8 V supply leads to a thrust increase of up to 15%. Although higher voltages boost thrust, they also significantly raise power consumption, nearly doubling it at 16.8 V. Since the motor is rated for only 150 W, it is strongly advised not to exceed 14.8 V to avoid overloading the motor, as its long-term reliability has not been tested at higher voltages. Generally, using these motors with a 12.6 V supply is recommended for optimal and safe performance.

#### 7.2.3 Testing different propellers

**Fig. 23:**
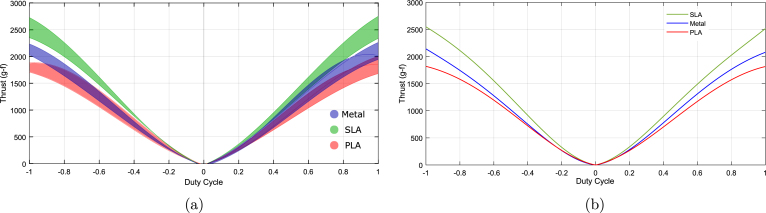
Thrust versus duty cycle: (a) all motors and propellers, (b) average thrust.

For this test, five different motors were randomly selected to limit the number of tests conducted. A lithium polymer battery powered the motors, as it closely resembles the actual vehicle setup. Fig. 23(a) presents a combined plot for all five motors with different propellers. The shaded region in the plot represents the upper and lower bounds of the thrust curves for each propeller across all motors. The SLA-printed propeller consistently outperforms the other two due to its smoother surface finish and greater blade stiffness compared to the FDM-printed propeller. In contrast, the metal propeller exhibits different performance characteristics, likely because it is significantly heavier and requires more torque from the motor. It is important to note that the propeller blades were designed primarily for 3D printing, especially optimized for FDM printing, resulting in thicker blades than those optimal for metal. A metal propeller designed with lighter alloy and better optimization for the material’s properties would likely perform better. The SLA-fabricated propeller achieved an average peak thrust approximately 35% higher than the PLA propeller and 18% higher than the metal propeller. These results suggest that, for a DIY thruster as presented in this work, an SLA-printed propeller offers the best performance. However, the SLA propeller costs roughly 120 times more than the PLA propeller. Therefore, if cost is critical, PLA should be preferred; otherwise, SLA provides superior performance.

**Fig. 24:**
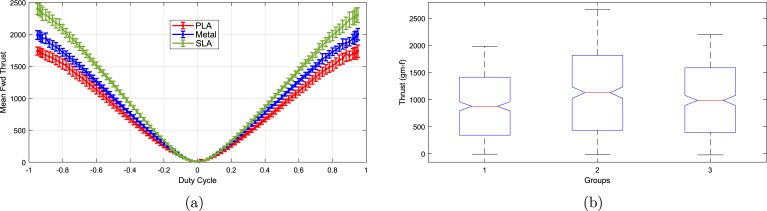
Statistical analysis of different propellers: (a) confidence intervals, (b) one-way ANOVA.

Fig. 24(a) shows the confidence interval plots for the propeller tests, illustrating the mean thrust response across the full duty cycle range for each material. The error bars are generally narrow and exhibit minimal overlap, particularly at duty cycles with absolute values greater than 0.2. This suggests that the differences in thrust performance between the materials are statistically significant across most of the operating range, highlighting the influence of material choice on thrust performance.

To validate this observation, a one-way ANOVA was performed, and the results are presented as a notched boxplot in Fig. 24(b), where groups 1, 2, and 3 correspond to PLA, SLA, and metal propellers, respectively. The non-overlapping notches in the plot further support the inference of significant differences among the group medians. The detailed ANOVA statistics are summarized in Table 10, which reports a P-value of $3.31\times 10^{-7}$—well below the standard significance threshold of 0.05. This strongly indicates that the null hypothesis (i.e., equal group means) can be rejected, confirming a statistically significant difference among the propeller types.

To further identify which group means differ, a Tukey’s HSD post hoc test was conducted, and the results are presented in Table 11. The confidence intervals in columns 3 and 5 of the table do not include zero, and all associated P-values are less than 0.05. These results confirm that the differences in thrust are attributable to the propeller material type, rather than random variation.

The average thrust coefficients ($k_{t}$) for the five motors tested in the pool with different propellers are as follows: $11.14\times 10^{-5}$ for SLA, $9.06\times 10^{-5}$ for PLA, and $9.0686\times 10^{-5}$ for metal propellers, where the thrust is expressed as $T_{h}=k_{t}\omega \left|\omega \right|$, with ω being the RPM. When expressed in radians per second, the thrust coefficients are 0.0102 for SLA and 0.0083 for both PLA and metal. The average constant relating torque to angular velocity is evaluated to be $6.077\times 10^{-7}$. Fig. 23(b) presents the nominal thrust versus duty cycle for all three propeller configurations. The duty cycle can be mapped to PWM values, with reverse thrust occurring between 980μs and 1490μs, and forward thrust between 1510μs and 2000μs. The equations for the average thrust as a function of the duty cycle for the three propellers are,

$$fs=2.7557\times 10^{3}x^{4}-7.3916\times 10^{3}x^{3}+6.4952\times 10^{3}x^{2}+657.4094x$$

$$fm=1.5270\times 10^{3}x^{4}-5.4835\times 10^{3}x^{3}+5.5064\times 10^{3}x^{2}+529.7514x$$

(1) $$fp=646.7142x^{4}-3.5325\times 10^{3}x^{3}+4.0322\times 10^{3}x^{2}+670.2431x$$

$$rs=2.2640\times 10^{3}x^{4}+7.1958\times 10^{3}x^{3}+6.9747\times 10^{3}x^{2}-506.3641x$$

$$rm=2.1959\times 10^{3}x^{4}+6.4177\times 10^{3}x^{3}+5.9965\times 10^{3}x^{2}-367.8235x$$

(2) $$rp=1.3273\times 10^{3}x^{4}+4.9531\times 10^{3}x^{3}+4.8909\times 10^{3}x^{2}-555.1603x$$

**Table 10:** One-way ANOVA statistics for different propellers.

Source	SS	df	MS	F	P
Groups	1.35068e＋07	2	6 753 376	15.11	3.30732e−07
Error	5.35013e＋08	1197	446 961.3		
Total	5.48519e＋08	1199

**Table 11:** Tukey’s HSD post hoc test on one-way ANOVA statistics for different propellers.

Group A	Group B	Lower limit	A–B	Upper limit	P-value
1	2	148.5883	259.3838	370.1793	0.0000
1	3	5.0989	115.8944	226.6899	0.0378
2	3	−254.2848	−143.4893	−32.6938	0.0068

Here, $f_{*}$ and $r_{*}$ represent forward and reverse thrust, respectively. The letters s, m, and p denote SLA resin, metal, and PLA propellers, while x is the duty cycle, ranging from 0 to 1 for forward thrust and from −1 to 0 for reverse thrust.

**Fig. 25:**
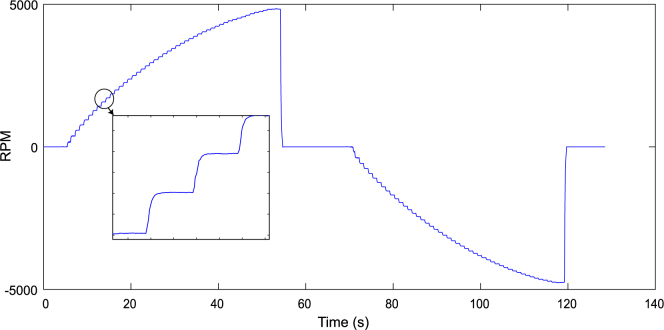
RPM v/s Time for Motor 9 with SLA propeller.

Data logged from the VESC6 was used to model the thruster’s angular velocity. This transfer function offers valuable insights into the thruster’s dynamic behavior, including rise time, responsiveness, stability, and overall control feasibility. Such a model supports researchers in developing or simulating control algorithms for similar thrusters. The RPM over time, extracted from the VESC6 log, is shown in Fig. 25.

A first-order fit based on the step changes in angular velocity yielded a rise time of 182.87ms. From this, the step response model for the thruster’s angular velocity was deduced as follows:

$$\frac{\omega_{d}}{\omega_{i}}=\frac{1}{0.0833s+1}$$

However, since the current system lacks a feedback loop for angular velocity, control is performed in an open-loop manner using PWM commands sent to the ESC.

Table 12 summarizes the upper and lower bounds, as well as the nominal values for the thruster based on the voltage tests. Meanwhile, Table 13 presents the corresponding bounds and nominal values based on tests with different propellers.

**Table 12:** Peak max, min and average thrust values at different voltages.

Parameter−>	Thrust (g–f)	Current (A)	Voltage (V)	Power (W)
	(F)/(R)	(F)/(R)		(F)/(R)
Peak max	1901/1871	21.9/21	12	276/265
Peak min	1537/1504	18/18	12	227/227
**Peak av.**	**1719**/**1687**	**20**/**19.5**	**12**	**251**/**246**

Peak max	2006/1767	21/21	12.6	264/264
Peak min	1556/1538	20/20	12.6	252/252
**Peak av.**	**1781**/**1652**	**20.5**/**20.5**	**12.6**	**258**/**258**

Peak max	2056/2046	25/26	14.8	370/384
Peak min	1888/1847	23.87/24	14.8	353/355
**Peak av.**	**1972**/**1946**	**24.43**/**25**	**14.8**	**361**/**369**

Peak max	2363/2350	29/30	16.8	487/504
Peak min	2077/2043	27/28	16.8	453/470
**Peak av.**	**2220**/**2196**	**28**/**29**	**16.8**	**470**/**487**

**Table 13:** Peak max, min and average thrust values for different propellers and test conditions.

Parameter−>	Material	Thrust (g–f)	Current (A)	Power (W)
		(F)/(R)	(F)/(R)	(F)/(R)
Peak max		1981/1899	28.3/28	328.5/340
Peak min	PLA	1621/1641	25.4/25.5	282/284
**Peak av.**		**1801**/**1770**	**26.8**/**26.7**	**305.2**/**312**

Peak max		2200/2181	26/25.6	301/303.4
Peak min	AlSi10Mg	1920/1928	23.7/23.6	263.8/261
**Peak av.**	(Alloy)	**2060**/**2054**	**24.8**/**24.6**	**282.4**/**282.2**

Peak max		2658/2651	26.6/26.6	318.8/320
Peak min	ABS-like	2224/2320	24.5/24.4	272.3/271
**Peak av.**	(Resin)	**2441**/**2485**	**25.5**/**25.5**	**295.5**/**295.5**

#### 7.2.4 Conclusion and future work

This work represents a significant update to our previous OpenThruster design, incorporating more rigorous testing procedures, expanded performance evaluation, and practical enhancements to improve accessibility and real-world reliability. The resulting thruster offers numerous advantages, including ultra-low cost, ease of fabrication, design flexibility, and — most importantly — comprehensive performance characterization. By addressing the key limitations of existing designs — namely, the lack of open-source access and insufficient performance data — this work aims to make underwater propulsion more transparent, reproducible, and accessible to a wider community of researchers and developers.

Using affordable A2212 BLDC motors, we conducted performance tests across motors sourced from different vendors. While the experimental results with ten motors showed an 11% variation in thrust, statistical analysis showed the difference to be insignificant. Additionally, we assessed propellers fabricated using three distinct 3D printing methods: FDM (PLA), SLA (ABS-like), and SLM (AlSi10Mg). SLA-printed propellers demonstrated the best performance, attributed to their smoother surface finish and increased stiffness. This was further validated through statistical analysis, which confirmed that the observed differences in thrust arose from variations in propeller fabrication methods and their resulting material properties.

The low cost of the developed thruster significantly lowers the barrier for students and researchers, especially in developing countries. Although these thrusters may not be reliable commercial products, they serve as valuable enablers for initial steps into marine robotics. Future work will focus on testing the thruster’s reliability under static and dynamic conditions on a marine vehicle over the long term.

## CRediT authorship contribution statement

**Milind Fernandes:** Writing – original draft, Visualization, Validation, Methodology, Investigation, Data curation, Conceptualization. **Soumya Ranjan Sahoo:** Conceptualization, Supervision, Writing – review & editing. **Mangal Kothari:** Supervision, Writing – review & editing.

## Ethics statements

This work did not involve any human subjects or animal experiments.

## Funding

This research did not receive any specific grant from funding agencies in the public, commercial, or not-for-profit sectors.

## Declaration of Generative AI and AI-assisted technologies in the writing process

During the preparation of this work the author(s) used ChatGPT in order to improve the readability, grammar and language of the manuscript. After using this tool/service, the author(s) reviewed and edited the content as needed and take(s) full responsibility for the content of the published article.

## Declaration of competing interest

The authors declare that they have no known competing financial interests or personal relationships that could have appeared to influence the work reported in this paper.

## Footnotes

1 https://www.thingiverse.com/search?q=ROV+thruster.

2 FreeCAD v0.19/v0.20.3/v1.0. https://www.freecad.org/.

3 https://github.com/prusa3d/PrusaSlicer.

4 https://www.creality.com/products/ender-3-v2-3d-printer-csco.

5 SALOME Version 9.13. https://www.salome-platform.org/.

6 OpenFOAM Version 2406. https://www.openfoam.com/.

7 https://www.adafruit.com.
